# Identification of an Epigenetic Signature for Coronary Heart Disease in Postmenopausal Women's PBMC DNA

**DOI:** 10.1155/2022/2185198

**Published:** 2022-08-19

**Authors:** Xiao Zhong, Ziguang Song, Pingping Gao, Mingyang Li, Zhongping Ning, Xiang Song

**Affiliations:** ^1^Department of Cardiology, The Fourth Hospital of Harbin Medical University, Harbin, China 150000; ^2^Zhoupu Hospital, Shanghai University of Medicine and Health Sciences, Shanghai, China 200000

## Abstract

Menopause is accompanied with an increased risk of cardiovascular disease. DNA methylation may have a significant impact on postmenopausal women's development of coronary heart disease. DNA methylation alterations in peripheral blood mononuclear cells (PBMCs) from women with coronary heart disease and healthy controls were detected using the Illumina Infinium MethylationEPIC BeadChip platform in this work. We employed Sangerbox technology and the GO and KEGG databases to further study the pathogenesis of coronary heart disease in postmenopausal women. After that, we used functional epigenetic module analysis and Cytoscape to remove the hub genes from the protein–protein interaction networks. Five genes (FOXA2, PTRD, CREB1, CTNAP2, and FBN2) were the hub genes. Lipid accumulation, endothelial cell failure, inflammatory responses, monocyte recruitment and aggregation, and other critical biological processes were all influenced by these genes. Finally, we employed methylation-specific PCR to demonstrate that FOXA2 was methylated at a high level in postmenopausal women with coronary heart disease. To better understand coronary heart disease in postmenopausal women's molecular mechanisms, our study examine the major factors contributing to the state of DNA methylation modification, which will help discover novel diagnostic tools and treatment options.

## 1. Introduction

Cardiovascular disease (CVD) is a leading cause of death globally [1]. The cost of treating CVD will climb as the population ages and the increase of obesity and diabetes [2]. Menopause is a time of increasing cardiovascular risk in women, and postmenopausal women have a higher risk of cardiovascular disease than men of the same age [3]. Coronary heart disease (CHD), the main type of CVD, is the leading cause of mortality in women over the age of 65, surpassing the incidence of cancer, chronic lower respiratory illness, and diabetes [4].

Apart from these discrepancies in traditional risk factors, a range of clinical conditions unique to women, such as polycystic ovary syndrome, gestational diabetes, preeclampsia, autoimmune diseases, and early menopause, could increase the risk of CHD [5].Due to a high morbidity associated with postmenopausal CHD, much emphasis has been dedicated to the disease's pathological process. A vast majority of studies have demonstrated estrogen's protective effect on the coronary arteries [6, 7]. Intuitively, estrogens appear to postpone the formation of plaques and the repercussions of coronary artery disease in women [8]. However, two studies have found no cardiovascular benefit from hormone replacement therapy (HRT) during a 6.8-year follow-up period [9, 10]. In addition, women with HRT may have had an elevated risk of coronary artery disease and nonfatal ventricular arrhythmias within the first year of treatment [9]. Compared to placebo, HRT had no favorable effects on the cardiovascular system, and estrogen therapy alone also increased the risk of stroke [11].Currently, the function of estrogen and how it affects the cardiovascular system is still unknown. While estrogen levels in premenopausal women may not be more vulnerable to cardiovascular disease than males, this is not the only contributing element; therefore, further mechanisms should be found by additional investigation.

CHD is thought to be caused by a combination of genetic and environmental factors [12]. Study on heritable changes in gene activity or function without changes in DNA sequence, known as epigenetics, is of critical importance to all disease processes [13], including in cardiovascular disease and its associated diseases [14]. In epigenetics, most DNA methylation occurs through the attachment of an extra methyl group (CH3) from S-adenosyl methionine (SAM) to the C5 position of cytosine-paired-guanine (CpG) dinucleotide sequences, leading to 5-methylcytosine (MeC) (5mC) formation, which is usually responsible for controlling tissue-specific gene expression, genomic imprinting, and the inactivation of the X chromosome [15].The DNA methyltransferases 3b (DNMT3b), 3a (DNMT3a), and 1 (DNMT1) catalyze DNA methylation, which is reversed by Tet methylcytosine dioxygenases (TET1, 2, and 3) [16].

Evidence suggests that abnormal DNA methylation may contribute to coronary heart disease. Methylation of ATP binding cassette subfamily G member 1 (ABCG1) and ATP binding cassette subfamily A member 1 (ABCA1) has been associated with coronary heart disease [17–19]. In individuals with CHD, the PCSK9 gene expression and circulating blood protein levels are linked to promoter DNA methylation [20].IL-6 promoter hypomethylation, which results in its overexpression and systemic inflammation, has been linked to an increased risk of CHD [21].Atherosclerosis, the primary pathophysiology of CHD, is an inflammatory disease, as it is well documented [22]. In research on epigenetic regulation of genes associated with atherosclerotic plaque vulnerability, the imprinted gene PLA2G7, which encodes lipoprotein-associated phospholipase A2 (Lp-PLA2), has been seen as one of the most hypomethylated genes showing the upregulated expression in response to inflammation [23]. Atherogenesis of the vascular system also may be aided by ER alpha gene methylation-associated inactivation in vascular tissue [24]. In light of what has been said so far, the relationship between CHD and DNA methylation has been studied in a number of studies; however, postmenopausal women have received less attention. Thus far, just a research by Ramos and colleagues found that postmenopausal women with lower global DNA methylation had an increased risk of cardiovascular disease [25]. The biological process that is controlled by methylation that is not right needs to be studied more. The role of epigenetic factors in the etiology of coronary artery disease in postmenopausal women needs to be looked into more.

We pooled and analyzed data from women with coronary heart disease and their equivalents, as well as data on differential DNA methylation, as part of our present work (from postmenopausal women with coronary artery disease (PC), healthy postmenopausal women (PCG), and young women (C)).The Illumina Infinium MethylationEPIC BeadChip was used to methylate the above volunteers. A total of 850 K probes were used to examine the promoter, enhancer regions, CpG island, and coding region.

## 2. Materials and Methods

### 2.1. Study Population and Sampling

This research used CHD clinical criteria to enroll four postmenopausal women with CHD, four normal postmenopausal women (using stenosis 50% cutoffs to assess whether or not they had coronary disease [26]), and four young women. Young women with a thickness of vessel intima-media thickness (IMT) of less than 1.0 mm were chosen for inclusion using Wang et al.'s [27] operation criteria. Menopause is described as the time period between the ages of 50 and 60 when menstruation ends for at least 12 months [28]. Patients who had cardiovascular and cerebrovascular diseases, such as cerebral hemorrhage, embolism, nonatherosclerotic vascular disease, myocardial infarction, cardiomyopathy, heart failure, congenital heart disease, valvular disease, diabetes, autoimmune diseases, severe liver and kidney disease, systemic infection, or any other inflammatory disease, cancer; those who have taken anti-inflammatory medicine or surgical therapy in the last 6 months; and those patients with familial hyperlipidemia, were all excluded from this study [29]. All the patients were chosen by the Department of Cardiovascular Medicine at the Zhoupu Hospital in Shanghai's Pudong New District and singed written informed permission. The hospital approved this research, and it was performed in accordance with the Declaration of Helsinki.

### 2.2. DNA Methylation Experiment

Following the instructions for extracting peripheral blood mononuclear cells (PBMCs), we took 8 ml of fasting peripheral blood from each research subject in the morning, from each sample, using Histopaque-1077 lymphocyte separation solution (Sigma, United States), and the experiment was completed in 4 hours. DNA was isolated from PBMCs by using the DNeasy Blood and Tissue Kit (Qiagen, Germany). The purity and concentration of DNA were estimated using Nanodrop 2000 (Thermo, Germany) and Quibt3.0. Then, 500 ng DNA of each sample was used in bisulfite convert using EZ DNA Methylation Kits (Zymo Research, United States), and the converted products were put into 850 K BeadChips in accordance with the manufacturer's guide and protocol (Illumina, United States).

### 2.3. Differential Methylation Position Analysis

Genome-wide DNA methylation was determined according to the manufacturer's instructions using the Illumina Infinium MethylationEPIC BeadChip (Illumina Inc., USA), which gave genome-wide coverage comprising >850 000 CpG methylation sites per sample. We selected probes with at least three beads from at least 10% of samples, non-CpG probes, multihit probes, probes situated on chromosomes X and Y, and (SNP-related probes) for future analysis. To determine the methylation level, the array data (.IDAT files) were processed in the ChAMP program of *R* (http://www.bioconductor.org/packages/release/bioc/vignettes/ChAMP/inst/doc/ChAMP.html). The beta value (*β* value), which was the ratio of the methylated probe intensity to the total probe intensity (sum of unmethylated and probe intensities plus constant, where is equal to 100), signified the methylation status of all probes. A DNA methylation score ranging from 0 (totally unmethylated DNA) to 1 (fully methylated DNA) (completely methylated) was used to express the *β* values. To account for the bias caused by the array's different bead types (peak-based correction normalization), the methylation data were normalized. Between the two groups, the average *β* values were compared. When the *p* value was lower than 0.01, |Δ*β*|>0.1, differentially methylated CpG sites were detected.

### 2.4. Distribution Analysis of Differentially Methylated Positions (DMPs)

According to 850 K array annotation, the DMPs were divided into several categories based on their distributions relative to genomic areas (3′ untranslated regions (UTR), intergenic region promoter, gene body) and CpG island regions (beach, shelf, island, or open sea). The promoter areas comprised the 5′ UTR, sections near 200 and 1500 bp of the transcription start site (TSS200, TSS1500), and the first exon.

### 2.5. Gene Ontology and Pathway Enrichment Analysis

We used the Sangerbox tools (http://www.sangerbox.com/tool), a free online platform for data analysis on genes related to DMPs, to conduct Gene Ontology enrichment analysis to filter overrepresented Gene Ontology words in three categories (biological processes, molecular function, and cellular component). Sangerbox methods were used to discover statistically significant enriched pathways from the Kyoto Encyclopedia of Genes and Genomes (KEGG) pathway enrichment analysis for genes associated with DMPs.

### 2.6. Construction of PPI Network and Related Analysis

PPI analysis was used to better understand the relationships between the two groups of genes in the development of CHD in postmenopausal women and unique biological pathways. We combined hypomethylation and hypermethylation genes to create PPI networks with minimal number of hypomethylation and hypermethylation genes. A PPI network was created using STRING 11.0 (https://string-db.org/). The cutoff threshold for the interaction score was set at 0.4. For further analysis, the findings were imported into Cytoscape 3.8.0. The hub genes were filtered using the CytoHubba software (http://apps.cytoscape.org/apps/cytohubba).

The total differential methylation site interactions were also analyzed using functional epigenetic modules (FEM, http://www.bioconductor.org/packages/3.1/bioc/vignettes/FEM/inst/doc/IntroDoFEM.pdf). The FEM method may be thought of as a functional supervised algorithm that leverages a network of gene relationships, such as a protein–protein interaction (PPI) network, to find subnetworks with a large number of genes being linked to a phenotype of interest.

### 2.7. Methylation-Specific Polymerase Chain Reaction (MS-PCR)

MS-PCR was performed on the bisulfite-treated DNA samples using primers specific for the methylated versions of FOXA2: forward 5′-GAGGGGTAGGTTAGTTCGGT-3′ and reverse 5′-AAAATCTAACCCCTCTAACTCCG-3′ FOXA2 methylated sequence and ACTB (all six actin proteins are made by this gene. Actins are a group of proteins with a high degree of evolutionary conservation) methylation sequence, forward 5′-TGGTGATGGAGGAGGTTTAGTAAGT-3′ and reverse 5′-AACCAATAAAACCTACTCCTCCCTTAA-3′. Both versions of FOXA2 and ACTB were amplified for 35 cycles at 60°C using the Hieff Unicon® Universal TaqMan multiplex qPCR master mix (Yeasen Biotech, China). MS-PCR products were separated into aliquots and examined on a 2% agarose gel before being stained with Ethidium Bromide (Sangon Biotech, China).

### 2.8. Statistical Analysis

Counting and quantitative analysis was performed using ImageJ program. To evaluate whether there were statistically significant differences between the means of three separate groups, the one-way analysis of variance is utilized. The comparisons were performed using *t*-test and pairwise *t*-tests. It was statistically significant when the *p* value was less than 0.05. GraphPad Prism 8 (GraphPad Prism Software Inc., California) was used to correlate the analysis and visual presentation for the statistical studies.

## 3. Result

### 3.1. Quality Control of the Methylation Array Data

**Figure 1:**
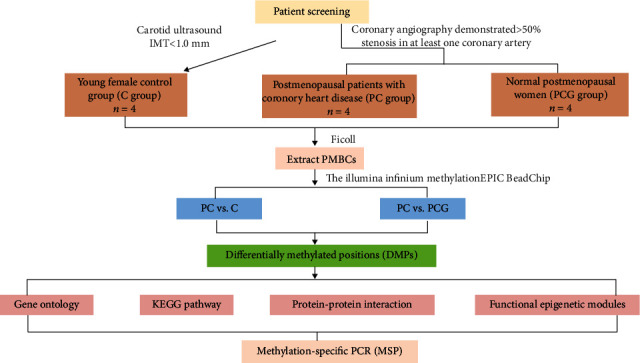
Flowchart of bioinformatics analysis. DMPs: differentially methylated positions.

**Figure 2:**
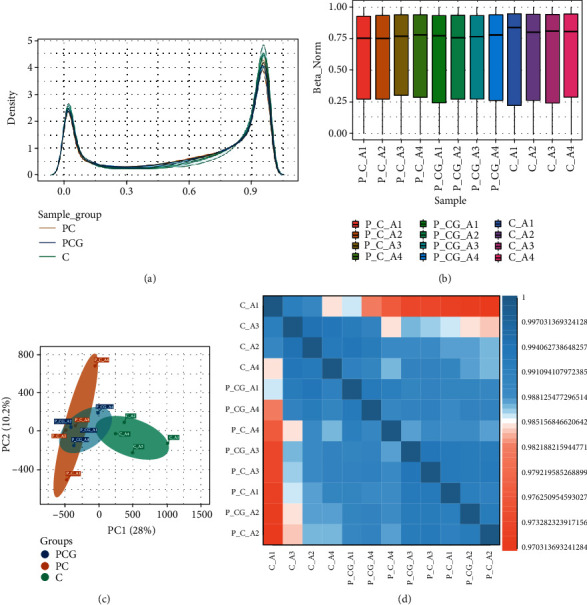
The methylation array data was subjected to quality control. (a) The density figure shows the distribution of the complete array probes for the PC, PCG, and C groups. (b) Boxplots of the twelve samples' *β* values. (c) A principal component analysis biplot of DNA methylation changes was used to highlight the data structure and sample link among the three sample groups. PC1 signifies the first principal component, whereas PC2 denotes the second main component. (d) A heat map illustrating three groupings of locations and samples that are differently methylation. The color shifts from red to blue, suggesting a strong to low correlation between the three samples.

The flowchart of the whole analysis is shown in Figure 1. The 12 subjects' genome-wide DNA methylation patterns were produced using the Illumina Infinium MethylationEPIC BeadChip. To quantify methylation at each locus, *β* values were used. Preprocessing and normalization of data from postmenopausal women with coronary heart disease and control samples were performed. The density distribution of the *β* values revealed a characteristic bimodal distribution (Figure 2(a)), with the first peak corresponding to low or unmethylated probes with a *β* value near 0 and the second peak corresponding to substantially or completely methylated probes with a *β* value near 1.Additionally, boxplots of the 12 individuals' *β* value distributions were obtained (Figure 2(b)).The results of the main component analysis on the three sets of samples are shown in Figure 2(c). The gap between samples was indicative of an individual gene's methylation pattern. Thus, the PC group's gene methylation profile was distinct from that of the other two control groups. A heat map of the relationships between the samples revealed that the research participants' gene methylation patterns varied (Figure 2(d)). The general distribution and concentration trend of the three data groups in the PC group and normal group were examined using the average *β* values of the three data groups. A heat map of the correlations among the samples and the PCA figure also supported that the sampling between groups was reasonable.

### 3.2. Identification of Significantly Differentially Methylated Sites

**Figure 3:**
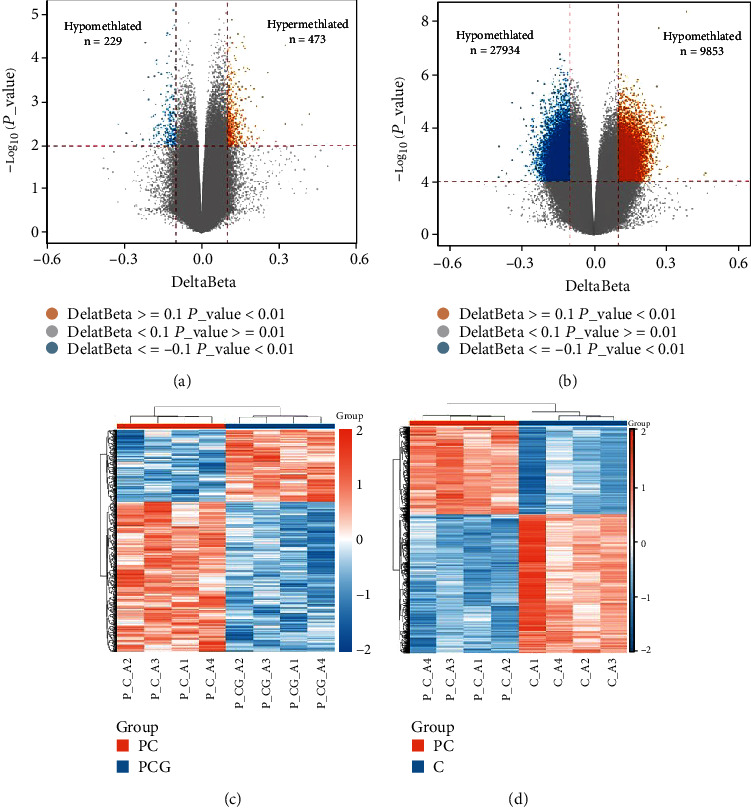
Methylation levels differed across groups. (a) A volcano plot of PC vs. PCG probe-level methylation. (b) A volcano plot of methylation at the probe level in PC vs. C. The graph depicts the link between the amount of the difference in values (*Δβ* values; *x*-axis) and the number of *p* values (negative log10 transformed *p* values; *y*-axis). A single probe is represented by each dot. Horizontal and vertical dashed lines represent the *p* = 0.01 and 10% methylation difference (|Δ*β*|=0.1) cutoffs, respectively. Hyper- and hypomethylated DMPs are represented by orange and blue dots, respectively. (c) A heat map of the top 5000 differentially methylated CpG sites in the PC versus PCG groups. (d) Heat map of PC vs. C group's top 5000 differentially methylated CpG sites. The heat map's orange color shows loci that have been hypermethylated, while the heat map's blue color shows loci that have been hypomethylated.

Following data preparation and quality control, 703,400 CpG sites were collected for further analysis after filtering. A total of 702 CpG sites were substantially differentially methylated with *p* < 0.01 and |Δ*β*| threshold>0.1 and comprised 473 hypermethylated and 229 hypomethylated sites in the PC group when compared to the PCG control (Figure 3(a)).Additionally, there were 37787 differential methylation sites, including 9853 hypermethylation sites and 27934 hypomethylation sites, across the PC and C groups (Figure 3(b)). Following that, we transformed the *z*-scores and carried out a heat map analysis on the whole set of differential methylation locations. We selected the top 5000 difference points for mapping or all mapping if the difference points were fewer than 5000. Figures 3(c) and 3(d) provide heat maps of all the substantially different methylation sites. Orange showed areas that were more hypermethylated, while blue showed areas that were less methylated. This indicated that PC patients and healthy people had different epigenetic landscapes.

### 3.3. Characteristics of Highly Differently Methylated Regions at the Genomic Level

**Figure 4:**
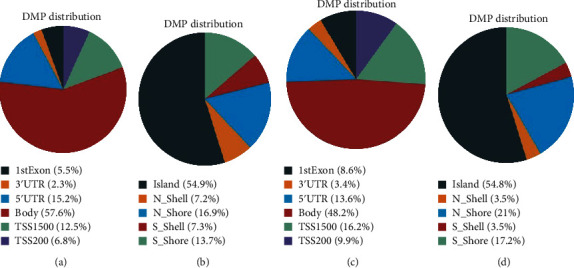
Differentially methylated positions are located on genomic CpG islands. (a) Distribution of methylated positions with respect to genomic regions in the PC vs. PCG group. (b) The distribution of methylated positions in the PC vs. PCG groups in relation to CpG island features. (c) Distribution of methylated positions with respect to genomic regions in the PC vs. C group. (d) The distribution of methylated positions in the PC vs. C groups in relation to CpG island features. 1stExon: first exon; TSS: transcription start site; TSS1500 and TSS200: 1500 bp and 200 bp downstream of the TSS, respectively; UTR: untranslated region. Island, probes located on CpG islands; open sea, probes not located on an island or annotated genes; shelf, probes located more than 2 k from CpG islands; and shore, probes located less than 2 kb from a CpG island.

We evaluated the CpG island for substantially different methylation sites in connection to their genomic positions. According to the CpG content, significant variations in methylated sites were identified between the PC and control groups. Figures 4(a) and 4(c) indicate the proportion of methylation in the 5/UTR, 3/UTR, transcription start site, exon, body, intergenic region, and intron for each group of postmenopausal females with CHD. The majority of substantially differentially methylated sites in postmenopausal women with coronary artery disease were located in the nonpromoter region. There was a great enrichment of DMPs situated on the CpG islands, whereas other DMPs were found in the open sea, beach, and shelf areas with low CpG island density (Figures 4(b) and 4(d).

### 3.4. Pathway Enrichment Analysis

**Figure 5:**
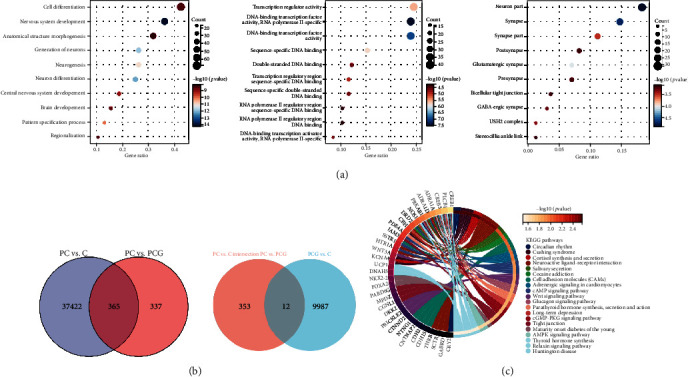
Analysis of differentially methylated locations (DMPs) for functional enrichment. (a) DMPs have enhanced Gene Ontology (GO) items in the categories of biological process (BP), molecular function (MF), and cell component (CC). Each category's top ten items were shown. (b) The intersection of differentially expressed loci is shown using a Venn diagram. (c) Kyoto Encyclopedia of Genes and Genomes (KEGG) pathway of DMPs. Cellular processes, environmental information processing, genetic information processing, human diseases, metabolism, and organismal systems are all subdivided into this section. The findings of the enrichment analysis for KEGG are shown in Figure 5(c). The top twenty items from each category were shown.

We intersected the difference loci obtained from the PC vs. PCG group with the difference loci obtained from the PC against the C group and acquired a total of 365 difference loci. In addition, discrepancies between the PCG and C groups were evaluated in order to rule out the influence of age. The difference between PCG and C loci was removed from the loci listed above (Figure 5(b)). We concluded with 353 distinct loci. More notably, these sites have the same methylation trend between the PC versus PCG and PC vs. C groups. According to the different loci, 234 genes were mapped. To further comprehend the functional implications of the DMPs, we used Gene Ontology and KEGG functional enrichment analyses. The most substantially enriched biological processes were cell differentiation, cellular developmental process, and nervous system development, according to functional annotation of these genes. Transcription regulator activity, DNA-binding transcription factor activity, and RNA polymerase II-specific and DNA-binding transcription factor activity were the most notably enriched molecular functions, and synapse, neuron projection, and neuronal parts were the most noticeably enriched cellular components (Figure 5(a)).

KEGG signaling network analysis was utilized to further analyze the signaling pathways associated with the differentially methylated genes between the PC and control groups. Numerous enriched KEGG pathways, including the cAMP signaling system, the Wnt signaling pathway, the cGMP-PKG signaling pathway, the AMPK signaling pathway, and adrenergic signaling in cardiomyocytes, have been implicated in postmenopausal women's coronary heart disease (Figure 5(c)).

### 3.5. PPI Network and Functional Epigenetic Modules

**Figure 6:**
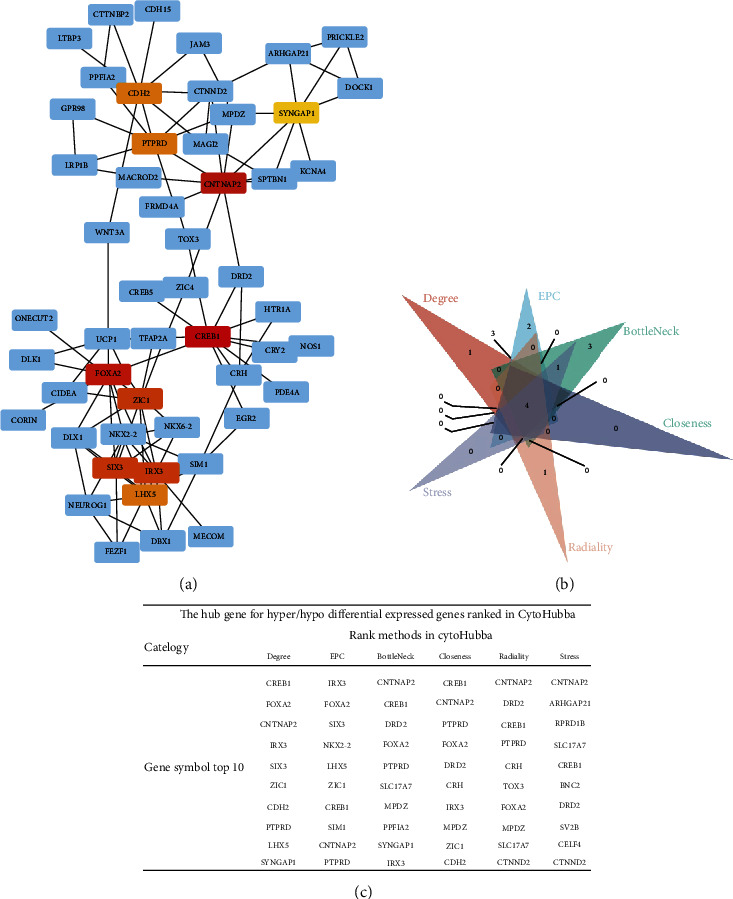
(a) Protein-protein interaction network of differentially methylated genes (DMGs). Disconnected nodes were hidden in the network. (b) This is a Venn diagram that shows where six of the best methods in CytoHubba overlapped hub genes. (c) CytoHubba-ranked hub genes for DMGs.

STRING identified 198 nodes and 174 edges in the PPI network. Cytoscape 3.8.0 was used to display the PPI network (Figure 6(a)), and the CytoHubba tool inside Cytoscape was used to pick the PPI network's hub node genes (Figure 6(b)). Four of the top ten hub genes identified in CytoHubba using six ranking methodologies were overlap hub genes (Figure 6(c)), including FOXA2 (forkhead box A2), CREB1 (CAMP responsive element binding protein 1), CNTNAP2 (contactin-associated protein 2), and PTPRD (protein tyrosine phosphatase receptor type D). Hypermethylation of FOXA2 and PTPRD was observed, but hypomethylation of CREB1 and CNTNAP2 was seen.

**Figure 7:**
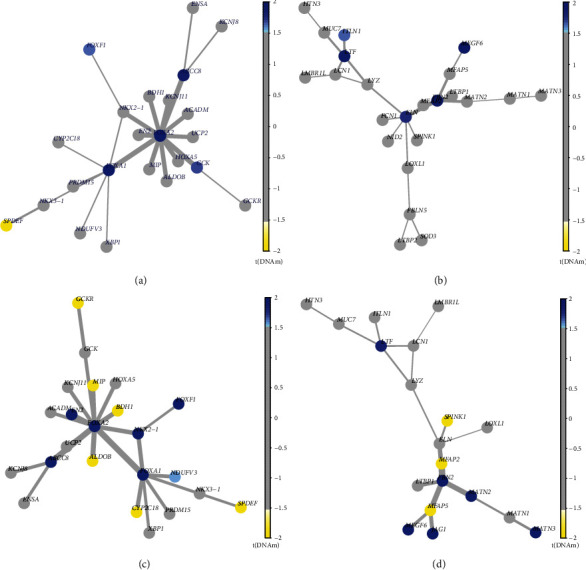
The core gene interaction network diagram was analyzed using a functional epigenetic module (FEM). In the FEM study, FOXA2 and FBN2 were shown to be the module's seed genes. (a) and (b) are the key gene results of PC vs PCG, while (c) and (d) represent the key gene results of PC vs. C. The protein–protein interaction (PPI) network defines the connection between each node in the diagram. The *T* value of differential methylation determines the node's color. It will be yellow to white if *T* is less than 1.5 and light blue to blue with gray in the center if it is larger than 1.5.

Using the FEM *R* program (http://www.bioconductor.org/packages/3.1/bioc/vignettes/FEM/inst/doc/IntroDoFEM.pdf), the FEM technique was used to further analyze the hub genes (also known as hotspots) of the methylated sites. Nine distinct functional modules were discovered in the PC versus PCG groups, and fifteen distinct functional modules were identified in the PC vs. C groups. The comparisons between the two groups included both FOXA2 and FBN2, which were notable as both of them were the module's seed genes, indicating that these genes played a critical role in the network. The network for the FOXA2 and FBN2 genes is shown in Figure 7. In comparison to control groups, FOXA2 was hypermethylated in the PC group (Figure 7(a)). Numerous members of the FOXA2 module interact with one another, including FOXA1, ABCC8, and FOXF1, all of which were hypermethylated in the PC group (Figure 7(c)). FBN2 was significantly more methylated in the PC group than in the control group (Figure 7(b)). Many members of the FBN2 module worked together, including MEGF6 and LTF, both of which were hypermethylated in the PC group (Figure 7(d)).

### 3.6. FOXA2 Hypermethylation Was a Possible Biomarker for Postmenopausal Coronary Heart Disease

**Figure 8:**
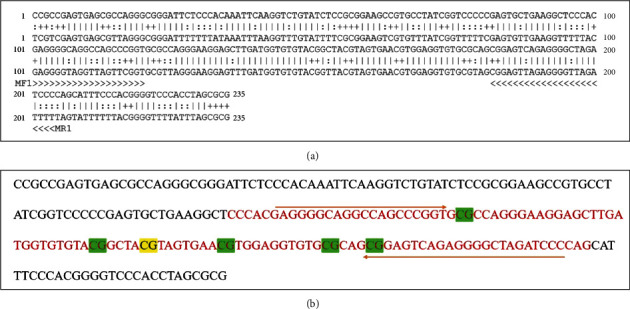
Chr20: 22566821-22567055, corresponding sequence of methylation islands. The above figures represent the gene sequence of CHR20: 22566821-22567055. (a) represents the process of designing methylation primers after bisulfite treatment. In (b), the red font represents the flanking sequence of the 850 K methylation chip detection site (upstream and downstream 50 bp), and the yellow background represents the CG16963144 detection site. The green background shows the CG sites that are used to detect MSPs. The orange arrow shows the primer binding site.

**Figure 9:**
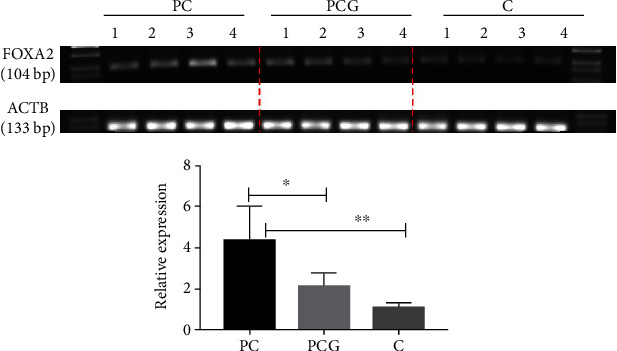
The FOXA2 promoter gene cg16963144 locus was expressed in three groups of patients.

Following confirmation by CytoHubba and FEM analysis that FOXA2 was a seed gene, MS-PCR was utilized to validate the gene's significant methylation sites. In order to develop primers, we focused on the chr20:22566821-22567055 coordinates of the methylation island in the UCSC library, where the cg16963144 site had the highest variation in chromosomal coordinates. Primers were created in MethPrimer 2.0 software for the cg16963144 location using the base sequence identified in the NCBI database (Figure 8(a)). Primers for the methylation internal reference gene, ACTB, were also created (Figure 8(b)). According to Figure 9, the PC group expressed more FOXA2 methylation products than the other two groups. Subsequent semiquantitative analysis revealed that FOXA2 methylation was considerably greater in the PC group than in the PCG group (*p* = 0.0380), and it was significantly lower in the C group (*p* = 0.0045).

## 4. Discussion

Epigenetic changes may be crucial in regulating the gene expression in molecular pathways and cellular processes associated with the pathophysiology of postmenopausal coronary heart disease [25]. DNA was taken from four of the postmenopausal women who have coronary artery disease and eight healthy women were controls (four postmenopausal women with CHD and four young healthy women). The DNA was then sent to a lab for analysis. To detect genome-wide methylation, the Illumina Infinium MethylationEPIC BeadChip was employed. The Illumina methylation chip (the Illumina Infinium MethylationEPIC BeadChip, 850 K methylation chip) is a next-generation DNA methylation chip built on the foundation of the original 450 K methylation chip. The chip had 91% of the original 450 K methylation chip and 413745 extra sites. It completely encompasses the promoter, coding region, CpG island, and enhancer regions of a gene [30].

In this study, we found that DNA methylation was involved in the process of CHD in postmenopausal women. The intersection of different groups was obtained through comparison. Finally, 353 different loci and 234 genes were selected for subsequent enrichment analysis and screening of key genes. We used GO and KEGG pathway analyses to infer the roles of differentially methylated genes. The functional annotation of these genes revealed that the most highly enriched GO terms were cell differentiation, cellular developmental processes, and nervous system development. It had been found that macrophages, vascular smooth muscle cells, and endothelial cells were engaged in all stages of atherosclerotic lesion formation [31]. Initially, we wondered why numerous gene methylation changes regulating the nervous system were found in PBMCs in postmenopausal women with coronary heart disease. Until recently, as reported in *Nature*, the nervous system regulated the progression of atherosclerosis through local remodeling of peripheral nerve fibers [32]. Moreover, genes enriched in this pathway were related to an increased risk of coronary heart disease, such as NRG1 [33], EGR2 [34], and NOS1 [35]. However, prior studies did not take methylation modification into account, and our analysis demonstrated that the methylation levels of key genes involved in these critical regulatory processes, such as PHOX2A, HOXD10, CREB1, and MYPN, were significantly increased. As a direct outcome of our findings, we hypothesized that changes in methylation levels may influence the differential expression of functional genes in women with coronary artery disease. To our knowledge, this is the first study to probe into the methylation profile of PBMCs from women who have coronary artery disease after menopause.

According to the KEGG database, the cAMP signaling route, the Wnt signaling system, the cGMP-PKG signaling pathway, and the AMPK signaling pathway all contribute significantly to the risk of coronary heart disease in postmenopausal women. The cAMP [36] and cGMP-PKG [37] signaling pathways have been implicated in the pathophysiology of coronary atherosclerosis. In the cardiovascular system, an activated AMPK signaling pathway had a protective effect [38]. The pathological process of atherosclerosis includes lipid buildup, endothelial cell failure, inflammatory responses, monocyte recruitment, and aggregation, when monocytes become tissue-resident macrophages and foam cells when they adhere to modified low-density lipoprotein. Under the intima, lipids accumulate, smooth muscle cells invade and multiply, and extracellular matrix accumulates. As a consequence of the restriction of the luminal lumen and reduced blood flow to the myocardium, chest pain, angina, and even myocardial infarction would occur. The Wnt pathway is involved in all phases of this process, from endothelial dysfunction to lipid deposition, and from early inflammation to the development of plaques [39].

CHD is the main cause of death globally, owing to the increased prevalence of CVD in postmenopausal women [40], and early prevention may help minimize morbidity and mortality. Thus, we should develop novel biomarkers to aid in the prognosis and treatment of coronary heart disease. In addition, important DNA is often methylated early in the disease, and looking for specific gene methylation that is linked to the disease has been used as a new diagnostic method. The methylation of the SEPT9 gene was the first biological marker recognized by the FDA for detecting DNA methylation in colorectal cancer screening [41]. DNA methylation detection, which is similar to it, may also be detected in the blood and may be useful indicators for CHD in postmenopausal women. As a result, we employed FEM and the CytoHubba software to find the hub genes in the PPI networks. Finally, FOXA2 was chosen as the seed gene, and its validity was confirmed using MS-PCR. FOXA2, also known as forkhead box A2, is a protein that is encoded by the FOXA2 gene and is found in humans. Transcription factor is implicated in embryonic development, the modulation of gene expression in differentiated tissues, and the establishment of tissue-specific gene expression, among other functions. Glucose homeostasis and fat metabolism are also regulated by it [42].At the time the study was completed, no link had been found between FOXA2 and coronary atherosclerosis. This is the first time that FOXA2 gene methylation has been found in postmenopausal women with coronary heart disease.

Furthermore, FOXA2, CREB1, CNTNAP2, and PTPRD are the four of the top 10 high hub nodes in the PPI network following gene overlap.FOXA2 serves as a validation gene as described above. Among these central genes, CREB1, also known as cyclic AMP (cAMP) responsive element binding protein 1, is a human protein encoded by the CREB1 gene. Even though postmenopausal people with CHD had hypomethylation of the CERB1 gene, it was found in the gene's body, and hypomethylation of the gene's body has been linked to transcriptional suppression [43]. CREB1 is involved in the regulation of apoptosis, and its overexpression is associated with atherosclerosis. The absence of the CREB protein in VSMCs, which makes them more susceptible to activation and death, is a common pathological response to vascular injury and may contribute to plaque development [44]; CNTNAP2 plays a key role in ischemic heart disease [45] and circulating lipid levels [46]; PTPRD has an effect on the onset and progression of CHD via acting on the glycine metabolism [47]. Additionally, FEM analysis showed a critical gene, FBN2, which was methylated in the promoter area in the case group, and displayed a decreased expression. In the absence of FBN2, p38 MAPK signaling is activated in an abnormally high manner, which in turn leads to an increase in MMP activity that disrupts elastic fiber production and degrades fibronectin [48]. In humans, fibronectin stimulates the creation of the protective fibrous cap, which prevents the rupture of plaques and vascular occlusion [49]. As a result, we hypothesized that these hub genes may be implicated in the pathological process in postmenopausal women with coronary artery disease, but more functional investigations are required to confirm this.

Our study still had certain limitations that should be addressed in future research. For instance, the study used a tiny number of participants because only 12 people were included in the study. The results must be replicated in a larger sample size for confirmation. We also need to perform MS-PCR or pyrosequencing for other genes that we have identified as methylation hotspots. It also lacked experimental evidence to support that aberrant methylation affected gene expression and function in postmenopausal women with CHD. Therefore, more molecular investigations are needed to confirm our findings.

In conclusion, we discovered that interactions between differentially methylated genes with distinct roles and signaling pathways were associated with the pathophysiology of postmenopausal women with coronary heart disease. FOXA2, CREB1, CNTNAP2, PTPRD, and FBN2 were the hub genes. This work improved conceptual and biological understanding on the pathophysiology of postmenopausal women with CHD. The identified genes and pathways in postmenopausal women with CHD still required more molecular-level studies both vitro and in vivo.

## Data Availability

The datasets used and/or analyzed during the current study are available from the corresponding author on reasonable request.

## Supplementary Materials

Supplementary Materials Table S1: primer sequences.
