# Supplementary material for: Identification of an Epigenetic Signature for Coronary Heart Disease in Postmenopausal Women's PBMC DNA
Source: Mediators Inflamm. 2022 Aug 19;2022:2185198. doi: 10.1155/2022/2185198 (PMC9417773; doi:10.1155/2022/2185198)
Supplement: Supplementary Materials — Table S1: primer sequences. [file 2185198.f1.docx]

Table S1. Primers sequences

| Genes | Forward | Reverse |
| --- | --- | --- |
| FOXA2 | 5′-GAGGGGTAGGTTAGTTCGGT-3′ | 5′-AAAATCTAACCCCTCTAACTCCG-3′ |
| ACTB | 5′-TGGTGATGGAGGAGGTTTAGTAAGT-3′ | 5′-AACCAATAAAACCTACTCCTCCCTTAA-3′ |
